# The proteasome deubiquitinase inhibitor bAP15 downregulates TGF-β/Smad signaling and induces apoptosis *via* UCHL5 inhibition in ovarian cancer

**DOI:** 10.18632/oncotarget.27219

**Published:** 2019-10-15

**Authors:** Shiho Fukui, Kazunori Nagasaka, Yuko Miyagawa, Ryoko Kikuchi-Koike, Yoshiko Kawata, Ranka Kanda, Takayuki Ichinose, Takeru Sugihara, Haruko Hiraike, Osamu Wada-Hiraike, Yuko Sasajima, Takuya Ayabe

**Affiliations:** ^1^ Department of Obstetrics and Gynecology, Teikyo University School of Medicine, Itabashi-ku, Tokyo 173-8605, Japan; ^2^ Department of Obstetrics and Gynecology, Graduate School of Medicine, The University of Tokyo, Bunkyo-ku, Tokyo 113-8655, Japan; ^3^ Department of Pathology, Teikyo University School of Medicine, Itabashi-ku, Tokyo 173-8605, Japan

**Keywords:** ovarian cancer, bAP15, TGF-β/Smad signaling, UCHL5, apoptosis

## Abstract

The ubiquitin-proteasome pathway plays an important role in the regulation of cellular proteins. As an alternative to the proteasome itself, recent research has focused on methods to modulate the regulation of deubiquitinating enzymes (DUBs) upstream of the proteasome, identifying DUBs as novel therapeutic targets in breast, endometrial, and prostate cancers, along with multiple myeloma. bAP15, an inhibitor of the 19S proteasome DUBs UCHL5 and USP14, results in cell growth inhibition in several human cancers; however, the mechanism remains poorly understood in ovarian cancer. Here, we found that aberrant UCHL5 expression predicted shorter progression-free survival (PFS) in a cohort of 1435 patients with ovarian cancer described in the Gene Expression Omnibus and The Cancer Genome Atlas databases. The subgroup of patients with *TP53* mutations was significantly more likely to exhibit poor PFS (*p* <0.001). Moreover, we found bAP15 could suppress *TP53*-mutant ovarian cancer cell survival by regulating TGF-β signaling through inhibiting UCHL5 expression and dephosphorylating Smad2, consequently inducing apoptosis. bAP15 (2.5 and 5.0 mg/kg) also exerted significant anti-tumor effect on nude mice bearing subcutaneous SKOV3 xenografts. As activated TGF-β signaling is involved in ovarian cancer progression, these findings suggest that UCHL5 inhibition offers potential opportunities for a novel targeted therapy against TGF-β-activated ovarian cancer.

## INTRODUCTION

Ovarian cancer, the eighteenth most common cancer in women worldwide with approximately 300,000 new cases reported in 2018 [1], comprises one of three common female malignant tumors arising in the genital tract and exhibits the highest mortality rate among female genital malignancies [1]. In general, the lack of early effective diagnostic methods results in the disease not being recognized until an advanced stage in 75% of cases; accordingly, disease mortality rate has remained fairly static at approximately 30% in recent years with short overall survival also driven by the emergence of resistance mechanisms [2]. The standard front-line therapy for advanced ovarian cancer consists of intensive debulking surgery followed by adjuvant chemotherapy. It is considered unlikely that in the near future a simple modification in chemotherapy agents combined with current conventional surgery will be sufficient to improve the poor prognosis associated with this disease.

Alternatively, advances in our understanding of the molecular mechanisms underlying ovarian cancer have identified several promising therapeutic targets including anti-angiogenic factors, poly ADP-ribose polymerase inhibitors, and immune-checkpoint inhibitors [3–5]. However, the identification of additional novel therapeutic targets is required to fulfill the promise of truly personalized care in ovarian cancer treatment.

The ubiquitin-proteasome system (UPS) is a highly specific and selective route for cellular protein degradation in all eukaryotic cells to regulate the fate of cellular proteins by striking a balance between the dynamic multifaceted post-translational modification processes of ubiquitination and deubiquitination of protein substrates [6–8]. Recently, the clinical approval of the proteasome inhibitors bortezomib, carfilzomib, and ixazomib has boosted new drug discovery programs targeting different components of the ubiquitin system [9–14]. In addition, a Phase I therapeutic clinical trial of bortezomib in combination with cisplatin has demonstrated that the treatment was well tolerated in patients with ovarian cancer [15]. Nevertheless, severe side effects and poor pharmacodynamic and pharmacokinetic properties of proteasome inhibitors, including bortezomib, have been reported [16]. Therefore, the identification of novel targets in the UPS will likely provide more effective single agent or combination therapies to better treat ovarian cancer.

The human genome encodes approximately 100 deubiquitinating enzymes (DUBs), which can be classified into six families: ubiquitin-specific proteases (USPs), ubiquitin carboxy-terminal hydrolases (UCHs), ovarian tumor proteases (OTUs), Machado-Joseph disease protein domain proteases, JAMM/MPN domain-associated metallopeptidases, and the monocyte chemotactic protein-induced protein family [17]. DUBs regulate multiple cellular processes including cell cycle control, DNA damage response and repair, apoptosis, chromatin modification, and response to external stimulations [17–20]. In mammalian cells, three different DUBs are associated with the 19S regulatory particle of the proteasome: USP14, UCHL5, and Rpn11. Both USP14 and UCHL5 constitute cysteine isopeptidases that cleave distal polyubiquitin chains and are suggested to hinder substrate degradation. Recent studies have identified DUBs including UCHL5 as novel therapeutic targets in breast, endometrial, and prostate cancers, neuroblastoma, and multiple myeloma [21–29], in part owing to their frequent overexpression in several types of carcinoma cells [17]. Thus, recent research has focused on methods targeting the regulation of DUBs upstream of the proteasome for cancer therapy rather than targeting the proteasome itself [17].

b-AP15 functions as an inhibitor of the USP14 and UCHL5 DUBs of the 19S regulatory particle. In contrast to 20S proteasome inhibitors, bAP15 blocks the deubiquitylating activity of both USP14 and UCHL5 to induce strong anti-tumor activity without affecting proteolytic activities of the 20S proteasome [25, 26, 28, 29]. Although the ability of bAP15 to induce cellular apoptosis in several kinds of carcinoma has been intensively investigated [25, 26, 28, 29], its effect on ovarian cancer remains unknown.

Among the different signaling pathways that may play a role in the transformation process of various ovarian tumor types, we consider that the effect of the *TP53* mutation on TGF-β signaling could play an important role in ovarian tumor progression as the latter is necessary for ovarian cancer cell proliferation [30]. Smad2/Smad3 represent direct targets of TGF-β receptor kinase 1 and mediate transcriptional regulation through their intrinsic ability to bind to DNA; their phosphorylation plays a crucial role in the pathogenesis of ovarian cancer [30]. Notably, UCHL5 has been reported to interact with Smad7 and potentially reverse Smurf-mediated ubiquitination of TGF-β receptor I [31]. However, the role of UCHL5 in the regulation of TGF-β signaling in ovarian cancer pathogenesis is still unclear.

In this study, we investigated the anti-tumor effect of the DUB inhibitor, bAP15, in advanced *TP53*-mutant ovarian cancer along with the underlying mechanism. In particular, we examined the ability of UCHL5 to de-ubiquitinate and stabilize Smad2/Smad3 in ovarian cancer cell lines, thereby promoting TGF-β signaling and contributing to the pathogenesis of *TP53*-mutant ovarian cancer. These data will contribute to determining the therapeutic potential of targeting UCHL5 in advanced *TP53*-mutant ovarian cancer.

## RESULTS

### UCHL5 genomic alterations in ovarian cancer

**Figure 1:**
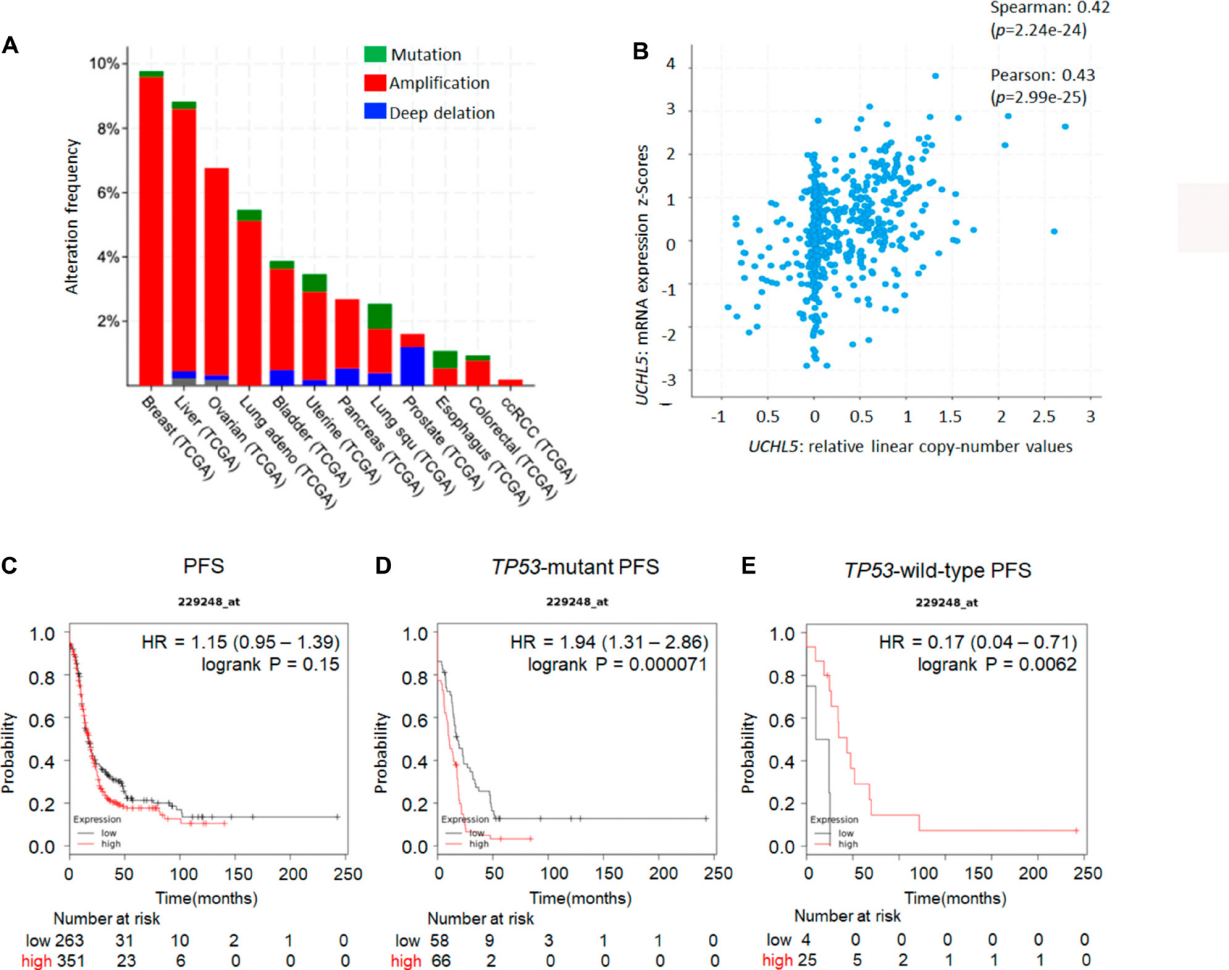
*UCHL5* expression across different organs and *TP53* status in ovarian cancer. (**A**) Overview of the genomic alterations in patients with cancer in the Cancer Genome Atlas TCGA database [38]. (**B**) To validate the correlations between the expression of *UCHL5* genes and the copy number alteration in an independent cohort, Affymetrix SNP 6.0 and RNA-Seq data generated by TCGA were accessed *via* the cBioPortal (http://www.cbioportal.org) [38]. The prognostic value of *UCHL5* mRNA expression downloaded from Gene Expression Omnibus and The Cancer Genome Atlas (Affymetrix HG-U133A, HG-U133A 2.0, and HG-U133 Plus 2.0 microarrays) in https://kmplot.com/analysis [39]. Affymetrix ID is 229248_at. Survival curves are plotted for patients with ovarian cancer. (**C**) the progression free survival (PFS) curve is in the right panel (*n* = 1435). Survival curves are plotted for patients with p53-mutated ovarian cancer. (**D**) the progression free survival (PFS) curve of ovarian cancer tissue with *TP53* mutation is plotted in the left panel (*n* = 483). (**E**) the progression free survival (PFS) of ovarian cancer tissue with wild-type *TP53* is plotted in the right panel (*n* = 84).

**Figure 2:**
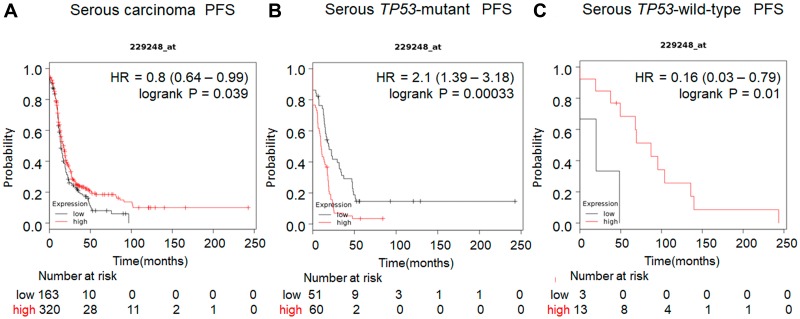
Prognostic value of UCHL5 expression in serous carcinoma of ovarian cancer patients available in https://kmplot.com/analysis. The prognostic value of *UCHL5* mRNA expression downloaded from Gene Expression Omnibus and The Cancer Genome Atlas (Affymetrix HG-U133A, HG-U133A 2.0, and HG-U133 Plus 2.0 microarrays) in https://kmplot.com/analysis [39]. Affymetrix ID is 229248_at. Survival curves are plotted. (**A**) the progression free survival (PFS) is plotted for patients with serous carcinoma (*n* = 1104). (**B**) The progression free survival (PFS) curve of ovarian cancer serous carcinoma tissue with *TP53* mutation is plotted (*n* = 470). (**C**) The progression free survival (PFS) of ovarian cancer tissue with wild-type *TP53* is plotted (*n* = 81).

Previous studies have demonstrated that the expression level of UCHL5 and associated clinical outcome vary among cancers including gastric, rectal, pancreas, esophageal, hepatocellular, and ovarian cancer [32–37]. Our analysis of several human cancers from the cBioportal database [38] revealed that gene alterations are frequent in *UCHL5* including amplification or mutation in several types of human cancer, specifically in 9.8% of breast cancer and 7% of high grade serous ovarian cancer (Figure 1A). Subsequent genomic analyses of 600 human high-grade serous ovarian cancers in The Cancer Genomics Atlas (TCGA) revealed *UCHL5* gene-containing amplicons across chromosome 1q31.1–1q31.2. Moreover, *UCHL5* copy number also significantly correlated with its mRNA expression level (R.0.42, *p* < 0.001, unpaired *t*-test) (Figure 1B). We also analyzed a database of gene expression and survival of 1435 patients with ovarian cancer, downloaded from Gene Expression Omnibus and The Cancer Genome Atlas (Affymetrix HG-U133A, HG-U133A 2.0, and HG-U133 Plus 2.0 microarrays) [39] for *UCHL5* mRNA expression, and generated survival curves (Figure 1C). The levels of *UCHL5* mRNA expression (Affymetrix ID: 229248_at) were not significantly different but were associated with poor progression free survival (PFS) (hazard ratio [HR] = 1.15; 95% confidence interval [CI] = 1.06-1.39; *p* = 0.15) in all patients with ovarian cancer, whereas significant difference was observed with those exhibiting *TP53*-mutant ovarian cancer (HR = 1.94; 95% CI = 1.31–2.86; *p* = 0.00071) (Figure 1D) with the inverse correlation observed in those with *TP53* wild-type ovarian cancer (HR = 0.17; 95% CI = 0.04–0.71; *p* = 0.0062) (Figure 1E). Furthermore, we investigated the histological analysis of serous carcinoma, as shown in Figure 2A. The levels of *UCHL5* mRNA expression were significantly associated with PFS (HR = 0.8; 95% CI = 0.64–0.99; *p* = 0.039). Moreover, a strong association was observed in *TP53*-mutant serous carcinoma (HR = 2.1; 95% CI = 1.39–3.18; *p* = 0.00033) (Figure 2B) but not in *TP53*-wild-type serous carcinoma (HR = 0.16; 95% CI = 0.03–0.79; *p* = 0.01) (Figure 2C) (Supplementary Figure 1).

### Cytoplasmic UCHL5 is a prognostic factor in advanced ovarian cancer

**Figure 3:**
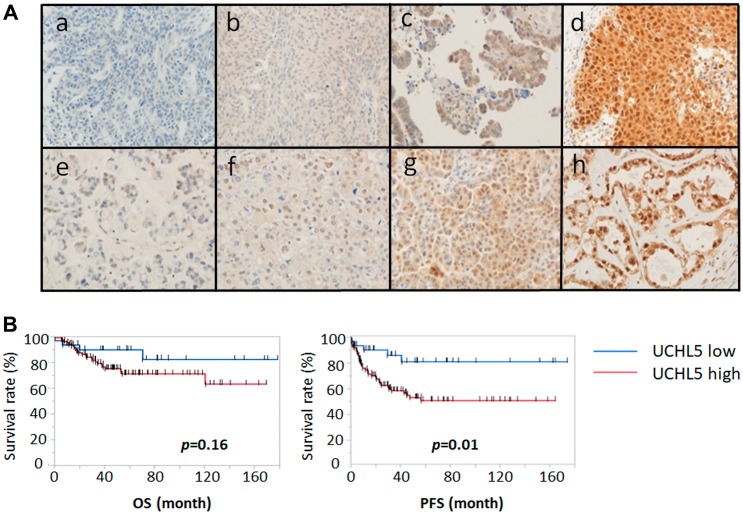
Immunohistochemical staining pattern of UCHL5 in ovarian cancer. We analyzed UCHL5 expression in 135 clinical ovarian cancer specimens using a tissue microarray. (**A**) UCHL5 cytoplasmic staining of serous carcinoma (upper panels **a**-**d**) and clear cell carcinoma (lower panels **e**-**h**) was scored according to intensity as either (**a**, **e**) 0, negative; (**b**, **f**) 1, weakly positive; (**c**, **g**) 2, moderately positive; or (**d**, **h**) 3, strongly positive. (**B**) Progression free survival (PFS) and overall survival (OS) were analyzed using Kaplan-Meier survival curves and log-rank test. The higher expression of cytoplasmic UCHL5 was significantly correlated with PFS decline. OS: HR 2.075 (95% CI: 0.721–5.975); PFS: HR 3.119 (95% CI: 1.231–7.899).

Immunohistochemical analysis of the tissue microarray specimens of ovarian cancers from 135 patients with advanced ovarian cancer treated at the Teikyo University Hospital from January 2003 to December 2012 was performed. The staining of UCHL5 was very weak in normal ovarian epithelium. Expression of UCHL5 was found in the majority of nuclei whereas its expression in the cytoplasm was various in ovarian cancer tissue. Figure 3A shows the representative results of tissues with low (Figure 3a, 3b, 3e, 3f) and high (Figure 3c, 3d, 3g, 3h) UCHL5 cytoplasmic expression in ovarian cancer tissue. Upper panels (Figure 3a–3d) show serous carcinoma; lower panels (figure 3e–3h) are clear cell carcinoma. UCHL5 cytoplasmic staining of ovarian carcinoma was scored according to its intensity as either (Figure 3a, 3e) 0, negative; (Figure 3b, 3f) 1, weak positive; (Figure 3c, 3g) 2, moderate positive; or (Figure 3d, 3h) 3, strong positive. Low and high expression of UCHL5 cytoplasmic staining was observed in 26.7% (36/135) and 73.3% (99/135) of patients, respectively (Figure 3A). High cytoplasmic UCHL5 expression was significantly associated with poor PFS (*p* = 0.01, HR 3.119, 95% CI: 1.231–7.899) for all patients with ovarian cancer, whereas there was no statistically significant difference in OS between the two groups (*p* = 0.16, HR 2.075, 95% CI: 0.721–5.975) (Figure 3B).

Next, we investigated the association between cytoplasmic UCHL5 expression and clinicopathological characteristics in patients with ovarian cancer. Univariate and multivariate analysis assessing prognostic factors were performed using Cox proportional hazards regression. Univariate analysis revealed that UCHL5 expression was significantly associated with FIGO stage, lymph node metastasis, and peritoneal dissemination, although we no significant association was found with histology and distant metastasis (Table 1). All variables associated with *p* < 0.10 on univariate analysis were included in the multivariate analysis; however, no significant independent prognostic factors were identified by multivariate analysis.

**Table 1:** Association of UCHL5 cytoplasmic expression and clinicopathological parameters

	UCHL5 expression					*p* value
	Number	Low		High		
	135	36		99		
			%		%	
Age (years)						
< 50	46	8	22.22	38	38.38	0.07
≥ 50	89	28	77.78	61	61.62	
Histology						
Serous carcinoma	54	9	25.00	45	45.45	0.08
Clear cell carcinoma	52	15	41.67	22	22.22	
Endometrioid carcinoma	27	8	22.22	19	19.19	
Mucinous carcinoma	17	4	11.11	13	13.13	
FIGO stage						
Early (stage I–II)	77	27	75	50	50.51	0.009
Advanced (stage III–IV)	58	9	25	49	49.49	
Lymph node metastasis						
pN0	115	34	94.44	81	81.82	0.04
pN1	20	2	5.56	18	18.18	
Distant metastasis						
M0	118	31	86.11	87	87.88	0.786
M1	17	5	13.89	12	12.12	
Peritoneal dissemination						
–	76	27	75	49	49.49	0.007
+	59	9	25	50	50.51	

### bAP15 inhibits cancer cell growth and induces apoptosis in a dose-dependent manner

**Figure 4:**
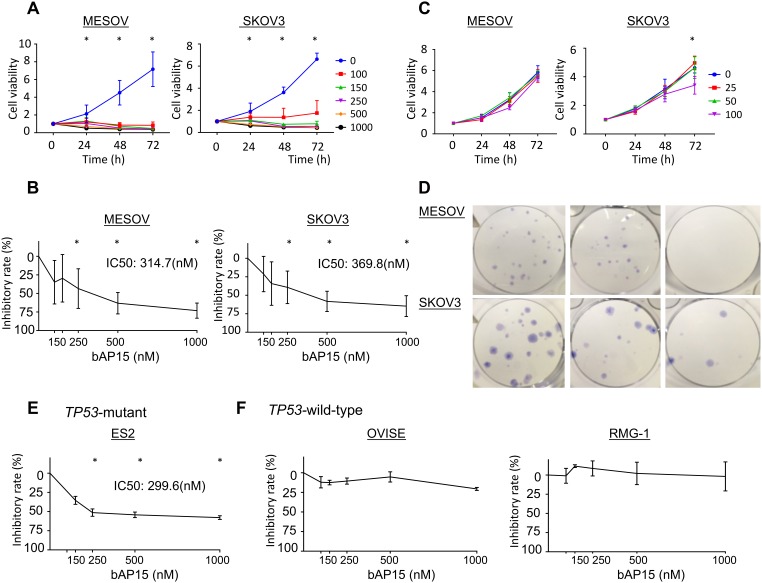
Inhibition of cell viability and colony formation of ovarian cancer cells by the DUB inhibitors bAP15 and IU1. (**A**) MESOV and SKOV3 cells were treated with various concentrations of bAP15 (0–1000 nM). Cell viability was detected by MTT assay. Three independent experiments were performed in triplicate; means ± SD are presented. ^*^ *p* < 0.05 versus each vehicle control. (**B**) The inhibitory rate was calculated from the cell viability of various concentrations of bAP15 (24 h). ^*^ *p* < 0.05 versus each vehicle control. (**C**) MESOV and SKOV3 cells exposed to IU1 (0–100 μM) for 9 and 11 days respectively, and (**D**) images of colony formation are shown. bAP15 but not IU1 significantly induced the inhibition of cell growth. (**E**) ES2 ovarian cancer cells with *TP53*-mutant were treated with various concentrations of bAP15 (0–1000 nM). Cell viability was detected by MTT assay. Three independent experiments were performed in triplicate; the means ± SD are presented. (**F**) OVISE and RMG-1 ovarian cancer cells with wild-type *TP53* were treated with various concentrations of bAP15 (0–1000 nM). Cell viability was detected by MTT assay. Three independent experiments were performed in triplicate; the means ± SD are presented.

We used bAP15 and IU1 as DUBs inhibitors and investigated their effect on the proliferation of ovarian cancer cells. bAP15 inhibited cell proliferation in a concentration-time dependent manner in both MESOV and SKOV3 *TP53*-mutant ovarian serous cell carcinoma cell lines (Figure 4A). The half-maximal inhibitory concentration (IC50) at 24 h was 314.7 nM for MESOV and 369.8 nM for SKOV 3 (Figure 4B). Conversely, proliferation was minimally suppressed even at 100 μM concentration of the USP14-specific inhibitor IU1 for these cells (Figure 4C). In addition, the colony formation assay demonstrated that the number of colonies was significantly decreased in cells treated with bAP15 compared with those in control cells (Figure 4D). Interestingly, bAP15 inhibited *TP53*-mutant ovarian cell lines ES2 in a concentration-time dependent manner (Figure 4E). In contrast, OVISE and RMG-1 did not show any inhibitory effect of bAP15 (Figure 4F), although it should be noted that these cell lines comprise different histologic types of clear cell carcinoma rather than serous carcinoma origin.

**Figure 5:**
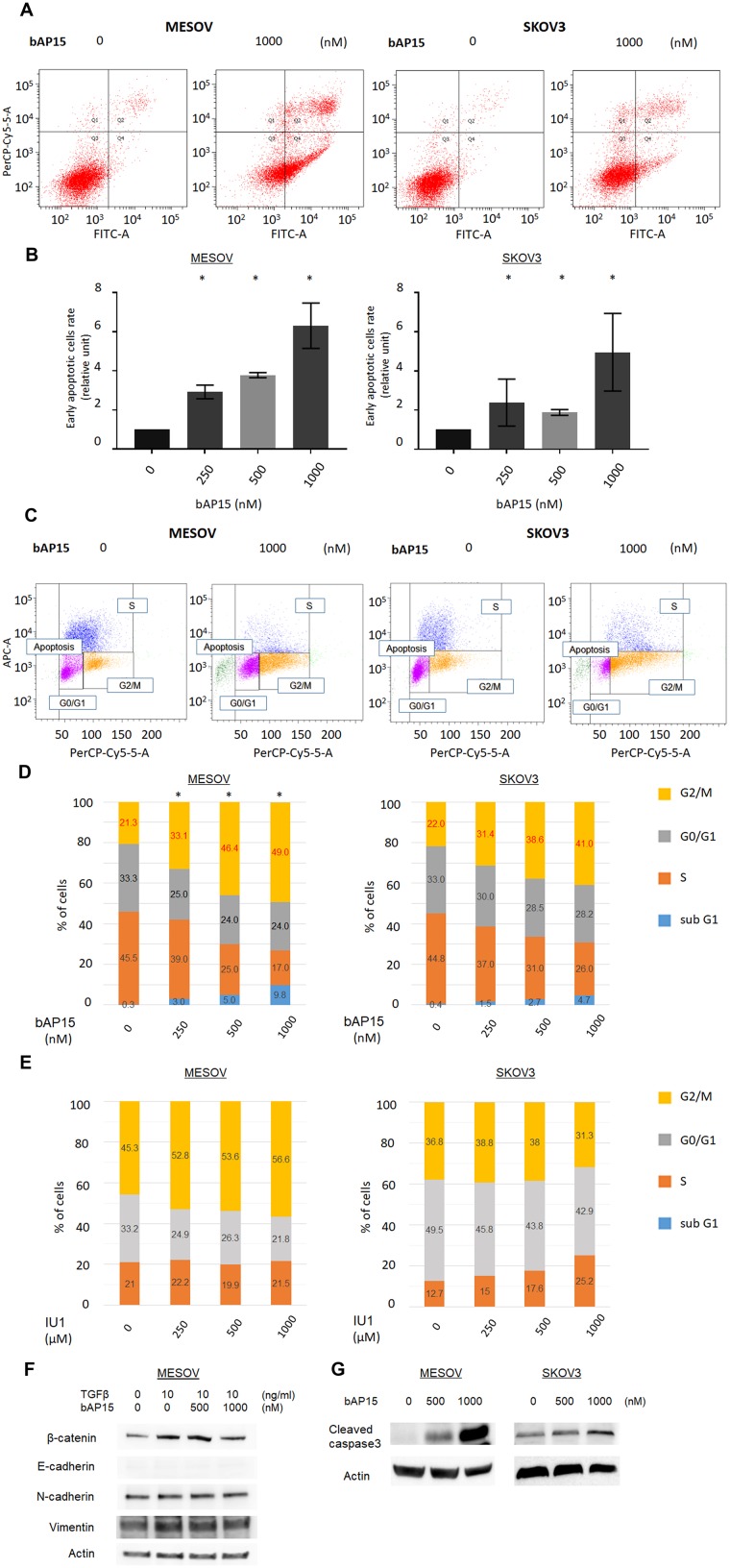
Proportion of apoptosis by bAP15. (**A**) MESOV and SKOV3 cells were treated with bAP15 for 18 h. The cells were harvested and stained by Annexin-V FITC/ propidium iodide (PI). Representative apoptosis analysis result is shown. (**B**) Quantification of the % of cells in early apoptosis (Q4) and late apoptosis (Q2). Relative ratios of apoptotic cells are shown. (**C**) MESOV and SKOV3 cells were treated with bAP15 for 18 h, and cell cycle status was analyzed by flow cytometry and PI staining. Representative apoptosis analysis result is shown. (**D**) Quantification of the % of cells in each quadrant treated with bAP15 for 18 h. Three independent experiments were performed in triplicate. ^*^ *p* < 0.05 versus each vehicle control. (**E**) Quantification of the % of cells in each cell cycle status treated with IU1 for 18 h. Three independent experiments were performed in triplicate. (**F**) Western blot analysis of E-cadherin and β-catenin in MESOV cell line after various concentrations bAP15 treatment with TGFβ-1 (10 ng/ml) stimulation for 30 min. (**G**) Western blot analysis of cleaved caspase 3 after various concentrations of bAP15 treatment in MESOV and SKOV3 cell lines.

Next, we investigated the apoptotic effect of bAP15 on MESOV and SKOV3 cells by flow cytometry using Annexin V. We found that bAP15 significantly induced apoptosis in these cells, with a greater effect on MESOV than SKOV3 cells (Figure 5A and 5B). Furthermore, cell cycle analysis following b-AP15 treatment of MESOV and SKOV3 cells revealed that bAP15 treatment resulted in a shift in the cycle distribution in both cell lines. Specifically, the G2/M phase cell cycle population of cancer cells was significantly increased after treatment of MESOV cells with bAP15 (Figure 5C). Moreover, Figure 5D shows that bAP15 induced G2/M cell cycle arrest in a concentration-dependent manner. In comparison, we observed no significant tendency for IU1 to induce the cell cycle arrest (Figure 5E).

To further investigate the mechanism of G2/M arrest and apoptosis induced by bAP15, we performed western blotting to observe the expression level of epithelial-to-mesenchymal transition related proteins following bAP15 treatment. As shown in Figure 5F, bAP15 attenuated the increased expression level of β-catenin induced by TGF-β1 (10 ng/ml) whereas no dramatic effect on the “cadherin-switch” was detected in the MESOV cell line, and cleaved caspase was induced by various concentration of bAP15 treatment in both MESOV and SKOV3 cell lines (Figure 5G).

### bAP15 inhibits cell growth *via* the TGF-β signaling pathway

**Figure 6:**
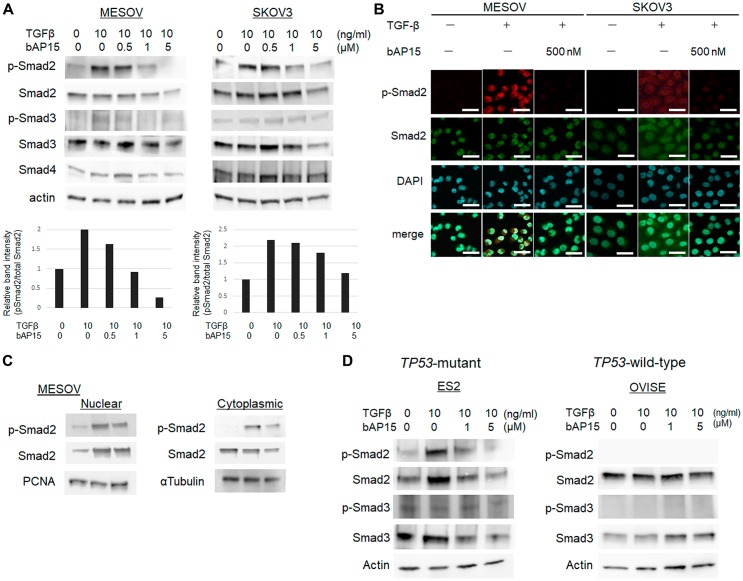
bAP15 induces downregulation of TGFβ-1 signaling *via* Smad2/3. (**A**) MESOV and SKOV3 cells were pre-treated with various concentrations of bAP15 (0–5 μM) for 1 h and then treated with TGFβ-1 (10 ng/ml) for 30 min. Phospho-Smad2, Smad2, Phospho-Smad3, Smad3, and Smad4 were detected by western blotting. β-actin was used as a loading control (upper panels). Quantification of the ratio of Phospho-Smad2 and Smad2 (lower panels). Independent experiments were performed in triplicate; means ± SD are presented. ^*^ *p* < 0.05 versus each vehicle control. (**B**) Immunofluorescence analysis. MESOV and SKOV3 cells were pre-treated with bAP15 (500 nM) for 1 h and then treated with TGFβ-1 (10 ng/ml) for 30 min. Expression of p-Smad2 was detected by immunostaining. DAPI was used for nuclei staining. Scale bar = 50 μm. (**C**) Subcellular fractionation analysis. Western blot analysis of nuclear and cytoplasmic extracts of MESOV cells were analyzed to detect the expression level of p-Smad2 and Smad2 in each fraction. PCNA was used as a loading control of nuclear extract and α-tubulin for cytoplasmic extract. (**D**) ES2 and OVISE clear carcinoma cells was pre-treated with various concentrations of bAP15 (0–5 μM) for 1 h and then treated with TGFβ-1 (10 ng/ml) for 30 min. Phospho-Smad2, Smad2, Phospho-Smad3, and Smad3 were detected by western blotting. β-actin was used as a loading control.

To further examine the effect of bAP15 on the TGF-β-induced signaling pathway, MESOV and SKOV3 cells were pretreated with increasing doses of bAP15 (0, 0.5, 1, or 5 μM) for 1 h, and then treated with TGF-β1 (10 ng/ml) for an additional 30 min. bAP15 attenuated the phosphorylation of Smad2 induced by TGF-β induction in a dose-dependent manner (Figure 6A), with a larger effect observed for MESOV than SKOV3 cells. The data were confirmed by immunofluorescence analysis (Figure 6B). Notably, both nuclear and cytoplasmic phospho-Smad2 was upregulated in MESOV cells upon TGF-β induction; in turn, these were dramatically downregulated following bAP15 treatment (Figure 6B, left panel). In contrast, the expression pattern of phospho-Smad2 by TGF-β induction was not dramatically changed in SKOV3 cells (Figure 6B, right panel). The results were confirmed using the cell fractionation assay in MESOV cells, wherein the nuclear and cytoplasmic expression levels of phospho-Smad2 were downregulated by bAP15 treatment (Figure 6C). Although *TP53*-mutant ES2 cells comprise histologic types of clear cell carcinoma, the expression pattern of phospho-Smad2 and Smad2 by TGF-β induction was significantly reduced following bAP15 treatment. In contrast, TGF-β induction was not dramatically changed in *TP53*-wild-type OVISE cells (Figure 6D).

### UCHL5 knockdown suppresses phosphorylation of Smad2 and bAP15 treatment suppresses the invasive capacity upon TGF-β signaling

**Figure 7:**
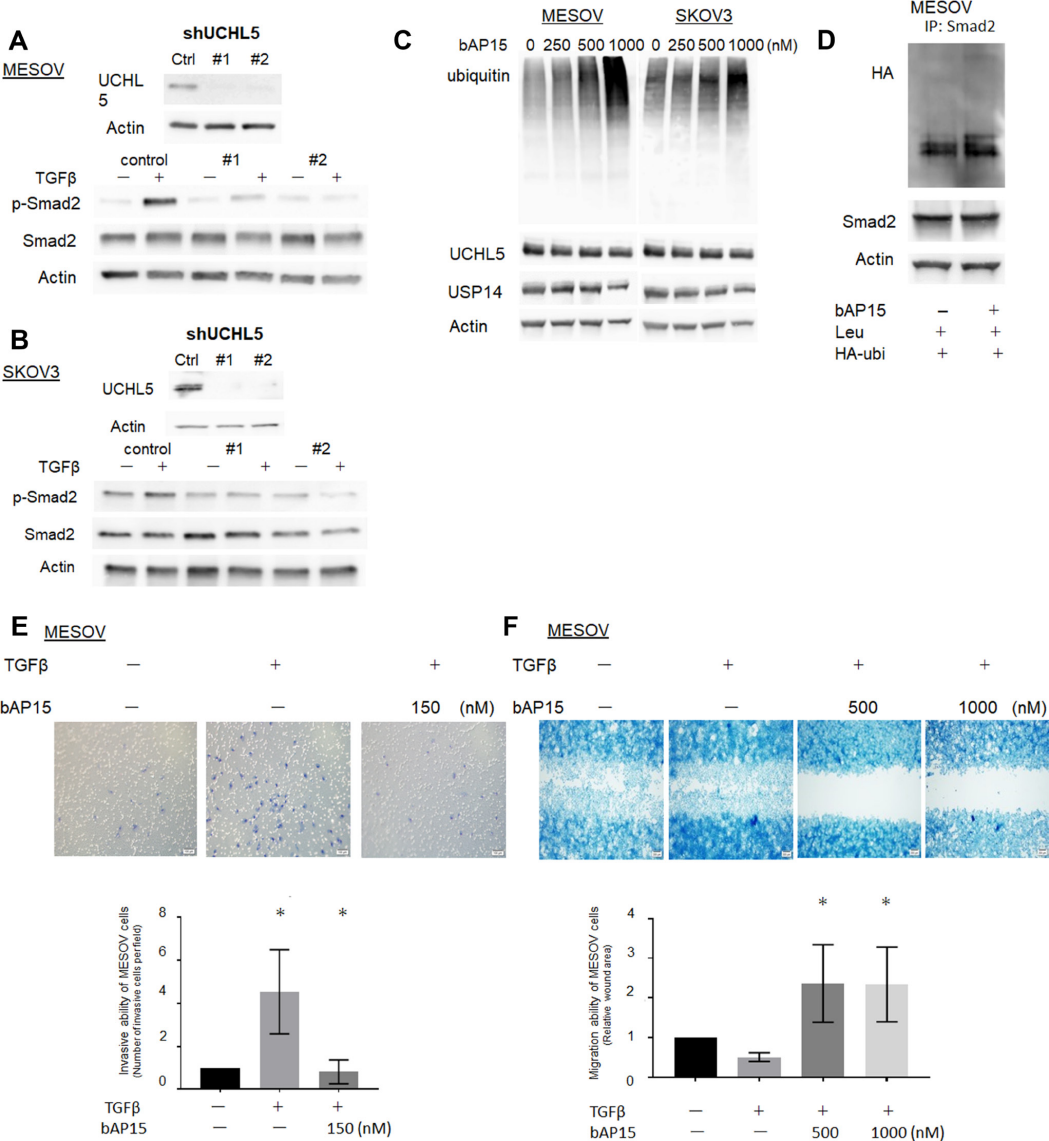
Knockdown of UCHL5 by shRNA suppresses phosphorylation of Smad2. (**A**) MESOV cells were co-transfected with scramble shRNA as a control and two different designs of UCHL5 shRNA (#1.2). UCHL5 was detected by western blotting. (**B**) MESOV of control and transfected shRNA were treated with TGFβ-1 (10 ng/ml) for 1 h. Phospho-Smad2, Smad2 were detected by western blotting. β-actin was used as a loading control. (**C**) Western blot analysis of ubiqutin, UCHL5 and USP14 after various concentrations of bAP15 treatment in MESOV and SKOV3 cell lines. Ubiquitin accumulated in conjunction with increased concentration of bAP15. (**D**) MESOV cells were transfected with HA-tagged ubiquitin (HA-Ub) for 48 h, treated with leupeptin (100 μM) for 2 h, and treated with bAP15 for 1 h. Cell lysates were subjected to immunoprecipitation with an anti-Smad2 antibody, followed by immunoblotting with HA-tag antibodies. Smad2 and β-actin were also analyzed by immunoblotting. (**E**) MESOV cells were seeded onto the top of a Matrigel Invasion Chamber for 24 h and removed from the upper chamber. The cells attached to the lower chamber were stained using the Diff-Quick reagent. Three independent experiments were performed in triplicate. ^*^ *p* = 0.035. (**F**) MESOV cells were seeded in the insert of the plate. Wounded cultures were incubated for 48 h and stained. Three independent experiments were performed in triplicate. ^*^ *p* < 0.05.

To determine whether the effect of b-AP15 on Smad2/Smad3 reduction occurs through inhibition of UCHL5, we investigated whether the shRNA-mediated depletion of UCHL5 stabilizes TGF-β/Smad signaling in MESOV cells. In the control, Smad2 phosphorylation was observed upon the addition of TGF-β, whereas the expression of phospho-Smad2 remained low following UCHL5 shRNA (#1, #2) treatment even upon TGF-β stimulation (Figure 7A). Similar results were observed in SKOV3 cells (Figure 7B), which demonstrated that UCHL5 knockdown stabilizes TGF-β/Smad signaling in both *TP53*-mutant ovarian cancer cell lines. Ubiquitin accumulated in conjunction with increased concentration of bAP15 in both *TP53*-mutant MESOV and SKOV3 cells, with a larger effect observed in the former (Figure 7C). Next, to investigate whether bAP15-mediated dephosphorylation of Smad2 was dependent on poly-ubiquitination of cellular proteins, we transfected MESOV cells with a plasmid encoding HA-tagged ubiquitin. Following subsequent culture of MESOV cells with bAP15, we examined changes in various proteins. Immunoprecipitation with anti-Smad2 antibody following the knockdown of UCHL5 by shRNA treatment in MESOV cells demonstrated an increased poly-ubiquitination of Smad2 (Figure 7D).

Furthermore, to assess the effect of bAP15 on cell invasion ability, we performed Matrigel transwell invasion assays using MESOV cells. Cell invasiveness and migration are related to pathophysiological processes such as cancer metastasis. These processes include changes in cell structure and cytoskeleton dynamics, expression of adhesion molecules, and activation of epithelial-mesenchymal transition signals. As shown in Figure 7E, MESOV cells were treated with TGF-β1 (10 ng/ml) then with bAP15 for 24 h. Although the invasive ability of MESOV cells was increased by the activation of TGF-β signaling, the invasive ability was suppressed in a bAP15 concentration-dependent manner (Figure 7E). Similarly, the results of wound healing assays demonstrated that the cell migration activity increased upon TGFβ signaling but was suppressed by bAP15 (Figure 7F). Taken together, these data reveal that the inhibition of UCHL5 by bAP15 stabilizes Smad2 and induces downregulation of TGF-β signaling.

### Antitumor activity of bAP15 *in vivo*

**Figure 8:**
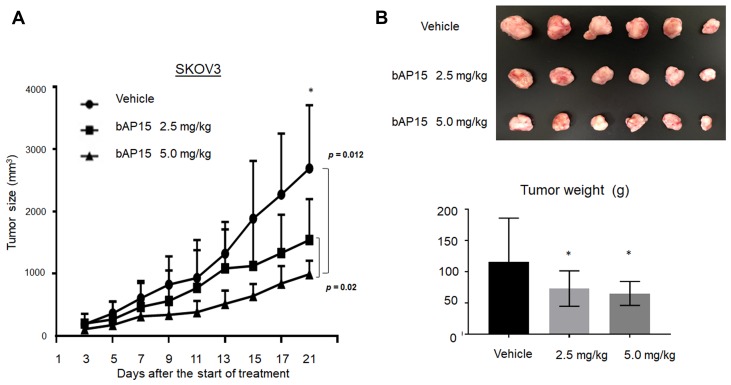
*In vivo* effect of bAP15 intraperitoneal treatment using SKOV3 xenograft mice models. (**A**) Subcutaneous tumor xenograft models in nude mice by using SKOV3 cells. bAP15 was dissolved in Dimethyl sulfoxide/Cremophor EL/NaCl (1:3:6). Each mouse was treated daily for three weeks with bAP intraperitoneal injection (2.5 mg/kg or 5.0 mg/kg) or vehicle. The mice were randomly assigned into three groups of 6 mice. ^*^ *p* < 0.05. (**B**) Pictures of tumors and the evaluation of tumor weight after the termination of treatment for 21 days. Comparison of tumor weight was evaluated by Student *t*-test. ^*^ *p* < 0.05.

The effect of anti-tumorigensis was examined using mice xenograft models of SKOV3. Two different doses of bAP15 (2.5 or 5.0 mg/kg) were injected daily into the peritoneum cavity of mice for three weeks. As shown in Figure 8, the administration of bAP15 significantly decreased the tumor size (Figure 8A) and tumor weight (Figure 8B) compared with a control (vehicle) in dose-dependent manner. This demonstrates the actual role of UCHL5 inhibition in *TP53*-mutant ovarian cancer *in vivo*.

## DISCUSSION

Our results suggested that UCHL5 activity is upregulated at a certain frequency in ovarian cancer. Notably, ovarian cancer comprises several different tumor types and different signaling pathways are involved in constitutive cell proliferation. For example, TGF-β signaling has been shown to regulate ovarian cancer development, whereas its disruption has been implicated in many cancers [30]. Initially, we hypothesized that a specific protein degradation system, the UPS, would constitute a key route to evaluate the critical targets in ovarian cancer progression. However, although targeting the UPS provides a new anticancer therapeutic strategy, the use of clinically available UPS-targeted inhibitors including lenalidomide and bortezomib is limited to the treatment of solid tumors [16]. Alternatively, DUBs serve as highly specific enzymes to regulate signaling pathways [7]. In particular, DUBs can reverse the effect of E3 ligases by removing ubiquitin from target proteins; moreover, DUBs are also involved in ubiquitin maturation, recycling, and editing [37], suggesting that ovarian cancer cells may rely heavily on the regulation of DUBs. Therefore, we further hypothesized that the levels of UCHL5, as a potential DUB to regulate TGF-β signaling, may correlate with clinical outcome in ovarian cancer.

The primary clinicopathological variable that contributes to poor prognosis in ovarian cancer is residual tumors in the peritoneal cavity following the primary treatment. Accordingly, peritoneal dissemination is a significant factor in prognosis. Some of these problematic tumors can be eliminated through platinum-based chemotherapy followed by primary debulking surgery; however, sites that have developed chemoresistance will exhibit gradual recurrence shortly thereafter [40]. Although growing numbers of oncoproteins and metastatic genes have been extensively characterized by comprehensive analyses, such as through the TCGA, many of these tumor-promoting proteins do not constitute good drug targets, which represents a major barrier to curing ovarian cancer. There is an urgent need, therefore, for alternative therapeutic approaches to neutralize cancer-promoting proteins. Thus, the investigation of specific UPS pathways to identify novel targets for personalized ovarian cancer treatment is warranted.

Our data revealed that cytoplasmic UCHL5 was upregulated in ovarian cancer, suggesting that cytoplasmic UCHL5 may serve as a marker of ovarian cancer progression. The TCGA database is a very useful resource to explore whether the expression levels of the genes near the *UCHL5* locus are in fact affected by copy number alteration. Figure 1B suggests that *UCHL5* mRNA is upregulated concomitant with its copy number alteration in response to growth stimulation in ovarian cancer. In addition, a cytoplasmic and nuclear UCHL5 staining pattern was observable in 135 patients with ovarian cancer (Figure 3A). Although the findings are limited owing to the rather small sample size in this study, notably, we found that cytoplasmic UCHL5 was significantly correlated with poor PFS in patients with characterized clinical data and long-term follow-up in a single institute. Moreover, the statistical analysis of the clinicopathological factors showed that the differentiation of ovarian cancer, FIGO stage, lymph node metastasis, and peritoneal dissemination were significantly associated with the expression level of cytoplasmic UCHL5. This raised the possibility that cytoplasmic UCHL5 may directly interact with TGF-β signaling in the cytoplasm before Smad2/3 enter the nucleus and participate in the regulation of cell proliferation and epithelial-mesenchymal transition-related genes.

TGF-β signaling is important in a wide range of cellular processes with regard to both normal physiological and pathological function. It is widely believed that TGF-β switches its role from tumor suppressor in normal cells to tumor promoter in advanced cancers, favoring invasiveness and metastasis depending on the tumor stage [30]. Numerous studies have implicated the potential of DUBs to regulate the TGF-β signaling pathway including USP4, USP11, USP15, CYLD, OTUB1, and UCHL5, which interact with Smads to regulate TGF-β signaling [38, 41–46]. In turn, Smads comprise direct targets of TGF-β receptor kinase and mediate transcriptional regulation through their intrinsic ability to bind to DNA. Consistent with this role, DUB function is frequently dysregulated in cancer [17]. In the present study, we confirmed that the blockade of UCHL5 activity by the DUB inhibitor bAP15 inhibited expression of phospho-Smad2/Smad3 in a concentration-dependent manner and induced apoptosis in ovarian cancer cells. In addition, we also used the pharmacological USP14 inhibitor IU1 to investigate the effect on cell growth in ovarian cancer cells. However, IU1 did not significantly affect cell growth in the present study, which suggested that USP14 is not critical in ovarian cancer and implied the lack of USP14-mediated androgen receptor signaling in ovarian cancer progression [22, 23].

In general, TGF-β signaling is frequently found to be activated in ovarian cancer. A recent publication showed that TGF-β signaling appears to play a role in ovarian physiology as well as acting as a tumor promoter that controls proliferation in ovarian cancer [30]. Although mutations in this pathway are rare in ovarian tumors, there are other mechanisms by which TGF-β is directly or indirectly associated with the promotion of ovarian cancer cell proliferation [30]. To investigate this mechanism further, we found that bAP15 could effectively suppress cell growth in the p53 null and mutant ovarian cancer cell lines SKOV3 (loss-of function *TP53*, nonsense mutation), MESOV (R282, hot spot mutation), and ES2 (S241F, missense mutation), respectively (Figure 4B, 4E). In contrast, the wild-type p53 ovarian cancer cells RMG-1 and OVISE did not show any significant effect of bAP15 (Figure 4F). Our observations were further reinforced through use of a data retrieval tool available at the KM plotter (http://kmplot.com/ovar) [39]. As p53 mutations are frequently observed in ovarian cancer (approximately 40–80% of all ovarian cancers), we evaluated the possibility that UCHL5 expression might be correlated with p53 status using the KM plotter. Notably, high expression level of UCHL5 was significantly correlated with poor PFS in patients with mutant p53 ovarian cancer (Figure 1D) whereas the opposite correlation was observed in patients with wild-type p53 ovarian cancer (Figure 1E). Although these data are insufficient to confirm the effect of p53 status on UCHL5 expression level and the effect of bAP15, we demonstrated that ovarian cancer cells null for or expressing mutant p53, but not wild-type p53, were growth inhibited by bAP15 (Figure 4B, 4E, and 4F). Obviously, further investigation toward delineating the mechanisms involved in the role of p53 mutation is essential, with emphasis on a “gain-of-function” mechanism related to DUBs rather than focusing on specific cross talk between TGF-β and the mutant p53 protein in ovarian cancer at the molecular level. Taken together, these findings suggest that the development of novel UCHL5-specific inhibitor might have a dramatic effect on TGF-β-activated metastatic advanced *TP53*-mutant ovarian cancer.

**Figure 9:**
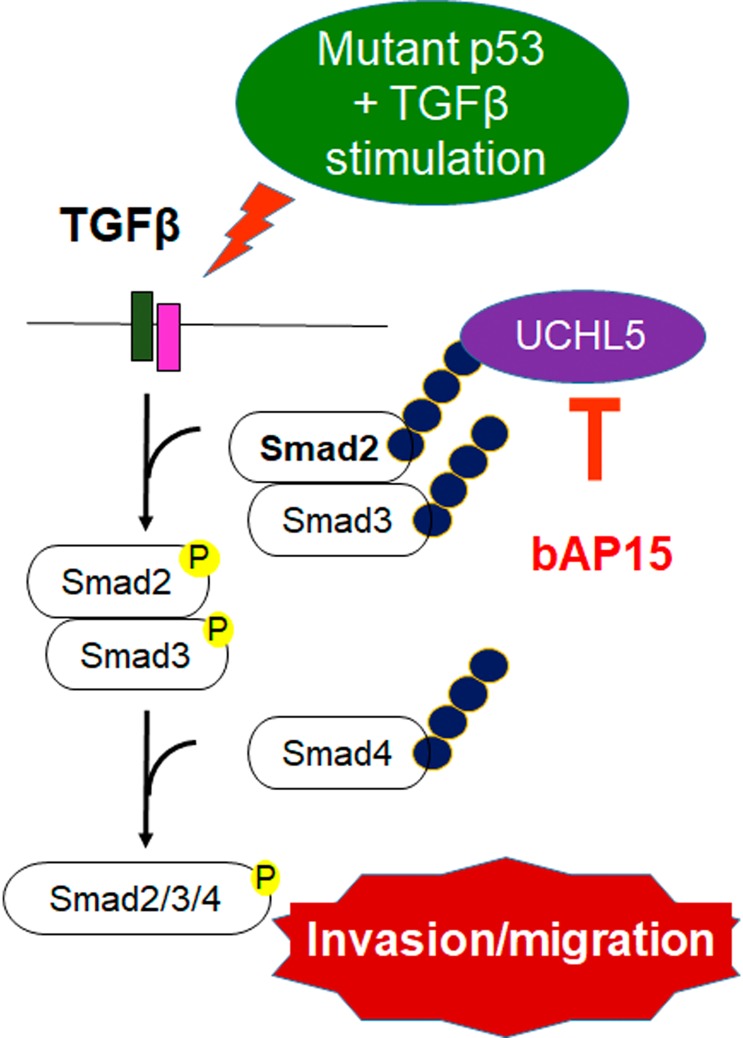
bAP15 inhibits the stabilization of Smad2/3 and aberrant TGF-β signaling in *TP53* mutant ovarian cancer. Schematic model demonstrating that the downregulation of UCHL5 by bAP15 or UCHL5 shRNA causes Smad2/Smad3 poly-ubiquitination, which promotes Smad2/3 degradation and attenuates aberrant TGF-β signaling in *TP53* mutant ovarian cancer.

In conclusion, our findings provide novel insight regarding UCHL5 and the TGF-β/Smad pathway in *TP53*-mutant ovarian cancer. To date, no data has been available regarding the prognostic role of UCHL5 in ovarian cancer. However, although various histological subtypes may not equally share TGF-β/Smad/UCHL5 axis function with those carrying *TP53* mutation, we consider that the concept may be applicable to the possibility of a critical role of UCHL5 at least in *TP53-*mutant serous carcinoma. In the present study, we revealed, for the first time, evidence of the clinical significance of cytoplasmic UCHL5 expression in ovarian cancer, and demonstrated that bAP15 significantly suppressed UCHL5 in *TP53*-mutant ovarian cancer cell lines in a dose-dependent manner through downregulation of the TGF-β/Smad signaling pathway (Figure 7). Notably, bAP15 had a growth inhibitory effect on SKOV3 and MESOV cells at a very low dose. Furthermore, we found that cytoplasmic expression of UCHL5 is significantly correlated with its regulation of ovarian cancer. Thus, targeting UCHL5 might serve as a promising novel therapy in advanced *TP53*-mutant ovarian cancer (Figure 9). Although UCHL5 inhibitors including bAP15 are currently being investigated for clinical use, future discovery of a novel *in silico* compound drug that specifically targets UCHL5 alone is warranted. Ongoing *in vivo* pre-clinical work will help elucidate the various contributions of UCHL5 toward TGF-β signaling, clarifying the ultimate outcome and potential benefits of inhibition of UCHL5 in patients with ovarian cancer.

## MATERIALS AND METHODS

### Materials

bAP15 (11324) was purchased from Cayman Chemical (Ann Arbor, MI). The USP14 inhibitor IU1 (662210) was obtained from EMD Millipore Corporation (Billerica, MA). UCHL5 shRNA plasmid (h) (sc-76797-SH) was purchased from Santa Cruz Biotechnology (Dallas, TX). HA-ubiquitin (Plasmid 18712) was purchased from Addgene (Cambridge, MA).

Antibodies were obtained as follows; anti-β-actin (sc-47778), anti-UCHL5 (sc-271002), anti-USP14 (sc-100630), anti-β-catenin (sc-7963), anti-Vimentin (sc-6260), anti-Ub (sc-166553), and anti-HA-probe (F-7) (sc-7392) (Santa Cruz Biotechnology), anti-E-cadherin (610181), anti-N-cadherin (610920)(BD Bioscience, San Jose, CA), anti-Smad2 (5339), anti-Phospho-Smad2 (3108), anti-Phospho-Smad2 (18338), anti-Smad3 (9523), anti-Phospho-Smad3 (9520), and anti-Smad4 (38454) (Cell Signaling Technology, Danvers, MA).

### Patients and analysis of tumor sample specimens

For generation of a tissue microarray for UCHL5 expression analysis, 135 clinical ovarian cancer specimens were obtained from the Teikyo University Hospital from January 2003 to December 2012. All patients had not undergone any treatment and ovarian cancer was confirmed by surgical and pathologic diagnosis. All patients provided written informed consent for the research use of their samples, and the collection and use of tissues for this study were approved by the Human Genome, Gene Analysis Research Ethics Committee at Teikyo University.

### Immunohistochemistry

Tissue samples were formalin fixed, embedded in paraffin wax, and cut into 4-μm sections. Paraffin sections were dewaxed in xylene and rehydrated through graded ethanol to water. Antigens were retrieved by boiling in 10 mM citrate buffer (pH 6.0) and endogenous peroxidase activity was quenched in methanol containing 3% hydrogen peroxide. The sections were incubated in phosphate buffered saline containing 3% bovine serum albumin to block nonspecific binding and incubated for 30 min with primary antibodies including anti-UCHL5 (1:100). We tested normal ovarian tissue as a positive control, and negative control tissues were incubated without primary antibodies. The sections were subsequently incubated with secondary antibodies and Envision FLEX (DAKO, Glostrup, Denmark). The antibody binding was visualized using a 3,30-diaminobenzidine solution (DAKO). After the sections were briefly counterstained with Mayer’s hematoxylin, the sections were dehydrated through a graded ethanol series and mounted.

UCHL5 cytoplasmic staining was scored according to its intensity as either negative (0), weakly positive (1), moderate positive (2), or strongly positive (3). UCHL5 immune expression was dichotomized into either low (score 0–1) or high (score 2–3), and the maximum score for each sample served for statistical analysis.

### Cell culture

The ovarian cancer cell lines MESOV, SKOV3, OVISE, RMG-1, and ES2 were obtained from the American Type Culture Collection (Manassas, VA) and cultured in McCoy’s 5A (Gibco, Grand Island, NY) containing 10% fetal bovine serum (Gibco) and maintained at 37°C in 5% CO_2_. The cell lines were authenticated by short tandem repeat (STR) profiling.

### Cell viability assay

Cells were seeded at the concentration of 1,000 per well in 100 μl medium in 96-well plates and treated with the indicated various concentrations of bAP15 and IU1. Cell Counting Kit-8 solution of 10 μl (Dojindo, Tokyo, Japan) was added and incubated for 3 h. Formazan dye was quantified using a microplate reader (BioTek, Winooski, VT) to measure the absorbance at 450 nm. Each experiment was performed in triplicate.

### Colony formation assay

Cells (1 × 10^2^ cells/well) were seeded in 6-well plates. After 24 h, the media were replaced with McCoy’s 5A containing the indicated concentration of bAP15. The cells were allowed to grow for 10 days, then fixed with 100% methanol for 10 min and stained with 0.5% crystal violet (Sigma-Aldrich, St. Louis, MO) for 10 min.

### Detection of apoptosis

Cells (5 × 10^4^ cells/well) were seeded in 6-well plates. After 24 h, the media were replaced with McCoy’s 5A containing the indicated concentration of bAP15 and IU1 and further incubated for 16 h. The cells were then washed with phosphate buffered saline and stained using Annexin V and propidium iodide (Annexin V-FITC Apoptosis Detection Kit I; BD Biosciences, San Jose, CA). Cell cycle distribution was analyzed by flow cytometry (FACS Canto II; BD Biosciences). Each experiment was performed in triplicate.

### Western blot analysis

Equal amounts of proteins were fractionated by sodium dodecyl sulfate-polyacrylamide gel electrophoresis and transferred onto a polyvinylidene difluoride membrane (Millipore, Bedford, MA). The membranes were blocked, primary antibody added, and incubated with secondary antibodies. Signals were detected using an Image Quant LAS 4000 Mini instrument (GE Healthcare, Wauwatosa, WI).

### Subcellular fractionation assays

Differential extraction of MESOV cells to obtain cytoplasmic and nuclear fractions was performed using the Nuclear/Cytosolic Fractionation Kit (Cell Biolabs, San Diego, CA) according to the manufacturer’s instructions.

### Cell cycle analysis

Cells (1 × 10^5^ cells/well) were seeded in 6-well plates. After 24 h, the media were replaced with McCoy’s 5A containing the indicated concentration of bAP15 and IU1 and further incubated for 16 h. The cells were stained with a BrdU Flow Kit (BD Biosciences, San Jose, CA, USA). Cell cycle distribution was analyzed by flow cytometry (FACS Canto II). Each experiment was performed in triplicate.

### Immunofluorescence

Cells were cultured on Chamber Slides^TM^ (Nunc, Rochester, NY) for 24 h. After treatment, cells were fixed with 4% paraformaldehyde for 30 min and blocked with 6% bovine serum albumin for 1 h. The cells were immunostained with a primary antibody for 1 h at room temperature and incubated with the fluorescent probe-conjugated secondary antibody for 1 h in the dark. Images were captured using a confocal fluorescence microscope (FV10i; Olympus, Tokyo, Japan).

### shRNA

We transfected UCHL5 shRNA plasmid DNA into the MESOV cell line cultured in 6-well plates using shRNA Plasmid Transfection Reagent (sc-108061; Santa Cruz Biotechnology) and shRNA Plasmid Transfection Medium (sc-108062; Santa Cruz Biotechnology). A scramble shRNA plasmid-A (sc-108060) was used as a negative control for the experiments. The cells were incubated for 2 weeks in medium containing puromycin (1 μg/ml). We also established stable control shRNA expression clones in these cell lines. The drug-resistant clones were further incubated in the medium with puromycin and tested for the knockdown effect by western blotting using the UCHL5 antibody.

### Immunoprecipitation

MESOV cells (1.5 × 10^6^ cells/well) were seeded in D100 plates. After 24 h, cells were transfected with HA-tagged ubiquitin (HA-Ub Plasmid 18712; Addgene) according to the transfection protocol. The cells were analyzed 48 h after transfection. Cell lysates were incubated with anti-Smad2 antibodies overnight at 4° C, followed by the addition of 20 μl Protein A Agarose Beads (9863; Cell Signaling Technology) and incubation for 3 h at 4° C. The immunoprecipitated complex was analyzed by immunoblotting.

### Wound healing assay

Cell suspension (5 × 10^5^ cells/well) was added onto 24-well plates in the insert in the plate (CBA-120; Cell Biolabs, San Diego, CA). Wounded cultures were incubated for 48 h and stained. Subsequently, 3 fields (40×) were randomly picked from each wound and visualized by microscopy to assess cell migration ability.

### Matrigel invasion assay

Invasion assays were performed according to the manufacturer’s instructions. Cells (1 × 10^5^ cells/well) in 0.5 ml of serum-free medium were seeded onto the top of a Matrigel Invasion Chamber (354481; Corning, Armonk, NY), and 0.75 ml of complete growth medium containing 10% fetal bovine serum was added to each well in the lower chamber. TGFβ and bAP15 were added to the top of the chamber. Following incubation for 24 h at 37° C, non-invasive cells were removed from the upper chamber, then the cells attached to the lower chamber were stained using Diff-Quick reagent (Sysmex, Kobe, Japan).

### Tumor xenografts in nude mice

Specific pathogen-free female nude mice (BALB/cAJc1-nu/nu) were purchased from CLEA Japan, Inc. (Meguro, Tokyo, Japan). Nude mice bearing SKOV3 tumor xenografts were established as described previously [47]. The mice were randomly assigned into three groups of six mice and received a daily peritoneal injection of bAP15 (2.5 mg/kg or 5.0 mg/kg) and vehicles. Tumor growth was measured daily and volume was calculated according to the formula ([major axis] × [minor axis]^2^) / 2.

### Statistical analysis

Survival was analyzed using the Kaplan-Meier survival plot and log-rank test. The HR with 95% CI were calculated. Statistical significance was determined using a Student’s t-test or one-way ANOVA in GraphPad Prism 6 software (GraphPad, San Diego, CA) and JMP 10 (SAS Institute, Tokyo, Japan), with *p* < 0.05 considered to be significant.

### Database retrieval

The survival information of patients with ovarian cancer was available from the Gene Expression Omnibus and The Cancer Genome Atlas (Affymetrix HG-U133A, HG-U133A 2.0, and HG-U133 Plus 2.0 microarrays). The prognostic value of *UCHL5* mRNA expression in ovarian cancer was assessed using the Kaplan-Meier plotter (http://kmplot.com/ovar). The *UCHL5* gene was used to screen the database; patient samples were divided into two groups (high vs. low expression) according to median expression, and the overall survival and PFS investigated using a Kaplan-Meier survival plot and log rank test, which were calculated automatically on the KM plot webpage. *p* value < 0.05 was considered to have statistical difference.

## SUPPLEMENTARY MATERIALS



## Abbreviations

CI: confidence interval
DUBs: deubiquitinating enzymes
HR: hazard ratio
OTU: ovarian tumor protease
PFS: progression free survival
PI: propidium iodide
UCH: ubiquitin carboxy-terminal hydrolase
UPS: ubiquitin-proteasome system
USP: ubiquitin-specific protease
