# Supplementary material for: The proteasome deubiquitinase inhibitor bAP15 downregulates TGF-β/Smad signaling and induces apoptosis via UCHL5 inhibition in ovarian cancer
Source: Oncotarget. 2019 Oct 15;10(57):5932–48. doi: 10.18632/oncotarget.27219 (PMC6800272; doi:10.18632/oncotarget.27219)
Supplement: Supplementary file 1 [file oncotarget-10-5932-s001.pdf]

The proteasome deubiquitinase inhibitor bAP15 downregulates TGF-β/Smad signaling and induces apoptosis *via* UCHL5 inhibition in ovarian cancer

SUPPLEMENTARY MATERIALS

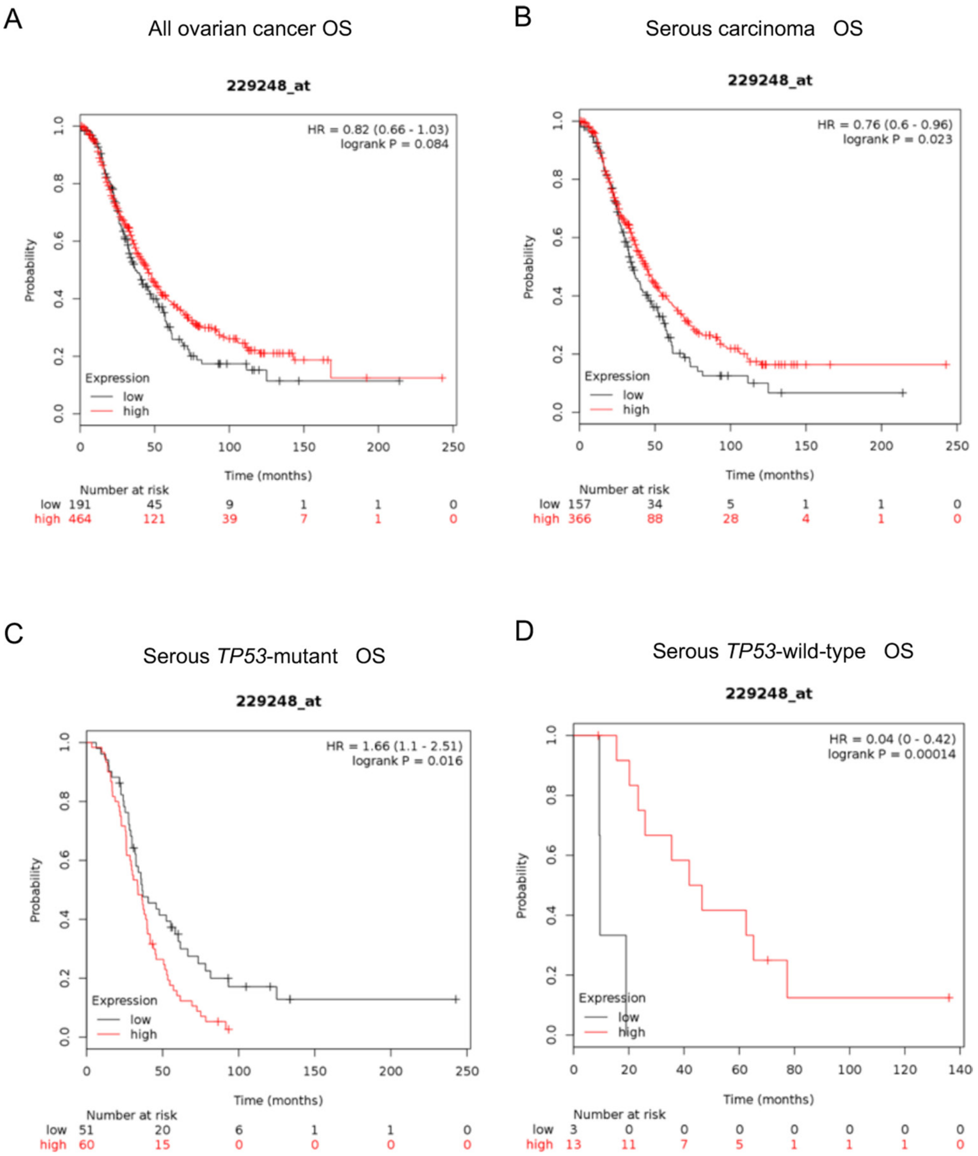

Supplementary Figure 1: Prognostic value of UCHL5 expression in patients with all ovarian cancer and serous carcinoma available in https://kmplot.com/analysis. The prognostic value of UCHL5 mRNA expression downloaded from Gene Expression Omnibus and The Cancer Genome Atlas (Affymetrix HG-U133A, HG-U133A 2.0, and HG-U133 Plus 2.0 microarrays) in https://kmplot.com/analysis [39]. Affymetrix ID is 229248_at. Survival curves are plotted for patients with p53-mutated ovarian cancer. (**A**) Overall survival (OS) curve of ovarian cancer ($n = 1657$). (**B**) OS curve of serous carcinoma of the ovary ($n = 1207$). (**C**) OS curve of serous carcinoma tissue with mutant TP53 ($n = 493$) and (**D**) OS curve of serous carcinoma tissue with wild-type TP53 serous carcinoma ($n = 91$).
